# Salvage high-dose chemotherapy for children with extragonadal germ-cell tumours

**DOI:** 10.1038/sj.bjc.6602724

**Published:** 2005-08-02

**Authors:** U De Giorgi, G Rosti, S Slavin, I Yaniv, J L Harousseau, R Ladenstein, T Demirer, G Dini

**Affiliations:** 1 Istituto Oncologico Romagnolo-Department of Oncology/Haematology, Santa Maria delle Croci Hospital, Ravenna, Italy; 2 Department of Bone Marrow Transplantation and Cancer Immunotherapy, Hadassah University Hospital, Jerusalem, Israel; 3 Department of Paediatric Haematology Oncology, Schneider Children's Medical Centre of Israel, Petah Tikva, Israel; 4 Department of Haematology, University Hospital, Nantes, France; 5 Department of Paediatric Haemato-Oncology, St Anna Children's Hospital, Vienna, Austria; 6 Department of Haematology/Oncology and Bone Marrow Transplant Unit, Ankara University Medical School, Ankara, Turkey; 7 Department of Paediatric Haematology Oncology, Institute G Gaslini, Genoa, Italy

**Keywords:** extragonadal germ cell tumour, high-dose chemotherapy, salvage therapy, children

## Abstract

We reviewed the European Group for Blood and Marrow Transplantation (EBMT) experience with salvage high-dose chemotherapy (HDC) in paediatric patients with extragonadal germ-cell tumour (GCT). A total of 23 children with extragonadal GCT, median age 12 years (range 1–20), were treated with salvage HDC with haematopoietic progenitor cell support. The GCT primary location was intracranial site in nine cases, sacrococcyx in eight, retroperitoneum in four, and mediastinum in two. In all, 22 patients had a nongerminomatous GCT and one germinoma. Nine patients received HDC in first- and 14 in second- or third-relapse situation. No toxic deaths occurred. Overall, 16 of 23 patients (70%) achieved a complete remission. With a median follow-up of 66 months (range 31–173 months), 10 (43%) are continuously disease-free. Of six patients who had a disease recurrence after HDC, one achieved a disease-free status with surgical resection followed by chemotherapy and radiotherapy. In total, 11 patients (48%) are currently disease-free. Eight of 14 patients (57%) with extracranial primary and three of nine patients (33%) with intracranial primary GCT are currently disease-free. HDC induced impressive long-term remissions as salvage treatment in children with extragonadal extracranial GCTs. Salvage HDC should be investigated in prospective trials in these patients.

Germ-cell tumours (GCTs) represent 3% of all paediatric neoplasms (Ries *et al*, 2003). The most common primary sites are the ovary (26%), the coccyx (24%), the testis (18%), the brain (18%), the mediastinum (4%) and the retroperitoneum (4%). Therefore, germ cell malignancies arising from extragonadal primary site represent nearly 50–60% of all GCTs in children (Gobel *et al*, 2000). Extragonadal primary location coupled with high serum levels of alpha-fetoprotein (AFP) (more than 10 000 ng ml^−1^) at diagnosis are the most unfavourable prognostic factors for these children (Mann *et al*, 1989; Marina *et al*, 1992; Baranzelli *et al*, 1999, 2000; Schneider *et al*, 2000; Lo Curto *et al*, 2003). Moreover, the prognosis of extragonadal GCT is significantly influenced by the histological differentiation, with the secreting GCTs (embryonal carcinoma, yolk sac tumour, choriocarcinoma) and immature teratoma having a higher risk of relapse after primary treatment than germinoma/dysgerminoma and mature teratoma (Gobel *et al*, 1999).

Conventional treatment for children with advanced GCT consists of cisplatin-based chemotherapy followed by surgical resection of residual disease, if necessary (Rescorla and Breitfeld, 1999). More than 80% of these children are cured with standard treatments (Mann *et al*, 2000; Kramarova *et al*, 2001). Very limited data are available concerning therapeutic options and long-term results of recurrent GCTs in children (Gobel *et al*, 2002).

High-dose chemotherapy (HDC) with haematopoietic progenitor cell support (HPCS) is a therapeutic option mainly investigated in adult patients with GCT either as first-line or salvage setting (De Giorgi *et al*, 2002). Preliminary experiences with HDC in children resulted in long-term remissions in those patients in whom a clinical complete remission (CR) could be achieved prior to HDC (Gobel *et al*, 2002). Subsequently, in children, HDC with HPCS has been mainly investigated as first-line treatment, or for consolidation therapy after primary standard-dose chemotherapy.

To better characterise the role of HDC with HPCS as salvage therapy for children with extragonadal GCT, the large database of the patients registered with the European Group for Blood and Marrow Transplantation (EBMT) was reviewed. This report describes the EBMT experience of salvage HDC in 23 children with extragonadal GCT.

## MATERIALS AND METHODS

### Data collection

The 23 relapse children analysed in this study belong to a cohort of 160 paediatric and adult patients with a diagnosis of extragonadal GCT, who have been registered with the EBMT from December 1987 to December 1999. The date of last follow-up was July 2003. Germ-cell tumours were classified as seminoma/germinoma, embryonal carcinoma, choriocarcinoma or mixed GCTs, mature or immature teratoma and yolk sac tumour according to the World Health Organization classification. Patients with histologically undifferentiated tumours with markedly elevated serum markers, who were treated according to GCT protocols, are included in this report. We reviewed the registration details of these patients. The reporting physicians were contacted and asked to provide further information on primary tumour site and extent of disease, histology, tumour markers, initial treatment, second-line chemotherapy, HDC drugs and toxicities, follow-up and data on possible secondary neoplasm. For data collection, a standardised questionnaire was sent to each centre. All patient data were obtained in an anonymous manner. Of 160 registered cases, 120 (75%) questionnaires were returned. Among these 120 patients, we analysed 23 cases of children with a diagnosis of extragonadal GCTs treated with salvage HDC, including three of nine cases with intracranial GCT, aged 15, 18 and 19 years, respectively. As this is a report of registry data, there are cases where information is incomplete, as indicated in the tables.

### Definitions

Tumour response was classified as follows: CR was defined as a complete disappearance of all clinical, radiological and biochemical evidence of disease, with normalisation of the tumour markers, beta-human chorionic gonadotropin (HCG) and/or AFP and/or lactate dehydrogenase (LDH), for at least a 1-month duration. A partial response (PR) was defined as a decrease in 50% or more of the sum of the products of perpendicular diameters of measurable disease, lasting at least for 1 month. If elevated markers were the only evidence of disease, a decrease of 90% or greater was required for a PR. In addition, reduction of the size of a tumour lesion and normalisation of previously elevated tumour markers was considered a partial remission with tumour marker normalisation (PR−), whereas a reduction ⩾50% in the sum of the perpendicular diameters of measurable disease plus a tumour marker decrease for at least 1 month, but without complete normalisation, was considered a marker positive partial remission (PR+). Stable disease (SD) was defined as a decrease <50% or an increase <25% in bidimensional tumour measurements or stable tumour marker levels. Progressive disease (PD) was defined as either residual lesions increasing in size or as the occurrence of new lesions and/or elevation of tumour markers at repeated controls.

### Patient characteristics

Details at diagnosis of the 23 patients are listed in Table 1. The median age was 7 years (range, 1–19). In all, 14 patients were males and nine were female. Nine patients had primary CNS (pineal/pituitary area), eight sacrococcygeal, four retroperitoneal and two mediastinal GCT. A total of 22 patients had a nongerminoma (three embryonal carcinoma, three yolk sac tumour, two immature teratoma, one choriocarcinoma, four CNS lesions with serum tumour marker increase, nine mixed GCT) and one germinoma. Most commonly, patients received cisplatin-based chemotherapy as first-line treatment. Table 1 summarises the characteristics of patients at diagnosis, primary treatment and response.

### Salvage treatment

In all, 13 patients received a second-line standard-dose chemotherapy given at first relapse/progression, while one patient received even a third-line standard-dose chemotherapy at second relapse/progression. Nine patients received salvage HDC in first-, 13 in second- and one in third-relapse situation. Nine (39%) patients received an induction and/or mobilising regimen before late-intensification HDC. The response to the last chemotherapeutic regimen before the induction regimen was CR in four cases, PR− in two and PR+ in three (Table 3). Then, the policy of EBMT centres was to use late-intensification HDC only in chemosensitive disease. In total, 14 (61%) patients were treated with upfront salvage HDC. The response to the last chemotherapeutic regimen before upfront HDC was PR+ in three cases, SD in one, PD in three, while six patients underwent HDC at the time of relapse after a previous CR or PR− (sensitive relapse) (Table 3). The salvage HDC regimens for extragonadal GCT patients were adapted based on the chemotherapeutic regimens given as initial therapy, and the salvage HDC protocols used in each centre for recurrent tumours in children, including drugs proven to be active in GCT. A total of 18 patients received one course of HDC, four underwent two courses; in one case three courses were given. The most commonly used HDC regimen was Carbopec, including carboplatin, etoposide and cyclophosphamide (*n*=7, 24%). High-dose chemotherapy regimens are listed in Table 2. Haematopoietic support consisted of peripheral blood progenitor cells (PBPCs) in 19 courses, autologous bone marrow transplantation (ABMT) in nine, both of them in one. Table 3 summarises the salvage HDC treatments and results.

### Statistical analysis

Descriptive statistics are presented as the median and range. Duration of followup and survival in this analysis were calculated based on the date of the first day of salvage chemotherapy until the date of last contact, if the patient was still alive, or until the date of death. Probabilities of disease-free survival and overall survival were determined using the Kaplan–Meier product limit method (Kaplan and Meier, 1958).

## RESULTS

### Toxicity

Toxicity data were fully assessable for 26 (90%) of 29 HDC cycles delivered. No treatment-related deaths occurred after HDC.

The median time to recovery of an absolute neutrophil count >500 *μ*l^−1^ and a platelet count >20 000 *μ*l^−1^, respectively, was 10.5 days (range, 6–22) and 10.5 days (range, 0–48). The median number of transfusions of red blood cell and platelet bags was 4 (range, 1–8) and 6.5 (range, 1–30), respectively. Fever occurred in 21 courses (81%), with an overall median duration of 3 days (range, 0–13). The number of HDC courses with episodes of clinically documented infections was 13 (50%).

The following nonhaematological side effects were the most relevant: grade ⩾3 stomatitis was reported in nine courses, grade ⩾3 pulmonary toxicity in two, grade 3 peripheral neurotoxicity in one, grade 3 anorexia in one and psychosis in one. Veno-occlusive disease occurred in two patients (pt. no. 8 and no. 16, Table 3), both received the HDC regimen, including carboplatin, etoposide, thiotepa and melphalan. Neither renal nor cardiac toxicity was observed. No patients developed myelodysplasia or secondary neoplasms after HDC.

### Response and survival

Overall, 16 (70%) patients achieved a CR. Of these patients, 14 obtained a radiological CR after HDC, while the other two with radiological PR− obtained a CR with post-HDC resection of residual masses and radiotherapy in one case. Results are presented in detail in Table 3.

**Figure 1:**
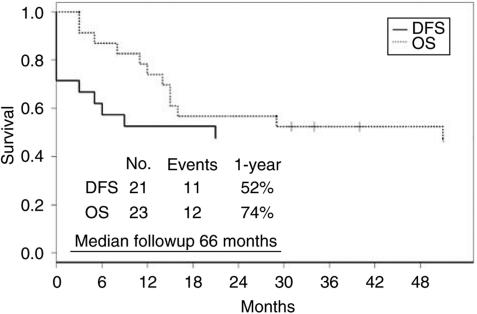
Disease-free and overall survival for patients with relapsing extragonadal GCT. Note: 21 of all 23 patients are assessable for disease-free survival (see Table 3).

The median follow-up period for all patients was 31 months (range, 3–173 months) and 66 months (range, 31–173) for surviving patients. Of 23 patients, 10 (43%) are continuously disease-free. Of six patients who had a disease recurrence after HDC, one underwent further surgery, chemotherapy and radiotherapy, and achieved a disease-free status. In total, 11 patients (48%) are currently disease-free (Figure 1).

The continuously disease-free status was achieved in four of nine patients (44%) with chemosensitive disease treated with late-intensification HDC, in four of six patients (67%) with sensitive relapse, who underwent upfront HDC, and in two of eight patients (25%) with PR+ or SD, or PD, receiving upfront HDC (Table 3).

Eight of 14 patients (57%) with extracranial primary GCT (five of eight patients (62%) with sacrococcygeal, two of four patients (50%) with retroperitoneal and one of two patients (50%) with mediastinal primary GCT) and three of nine patients (33%) with intracranial primary GCT are currently alive disease-free.

**Figure 2:**
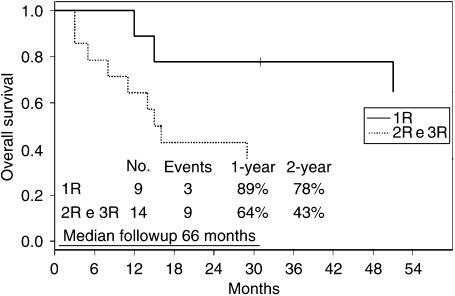
Overall survival with respect to the first (nine patients) and further relapses (14 patients).

Figure 2 illustrates the outcome of patients with extragonadal GCT, according to the time of relapse. The 2-year overall survival rates for first relapsing patients and second–third relapsing patients were 78 and 43%, respectively.

## DISCUSSION

Children with relapsing/progressing extragonadal GCT are characterised by a poor prognosis, with a salvage rate in the first relapse in the range of 40–45% (Marina *et al*, 1992; Baranzelli *et al*, 1999; Sawamura *et al*, 1999; Gobel *et al*, 2000; Mann *et al*, 2000; Schmugge *et al*, 2000).

According to extragonadal primary location, histology and the site of recurrence/progression, the salvage strategies are different. Standard-dose cisplatin- or carboplatin-based chemotherapy is the conventional treatment for most of these patients. In patients with local recurrence of sacrococcygeal GCT, the surgical complete resection represents the cornerstone of the salvage treatment, while chemotherapy is employed in recurrent inoperable and metastatic disease (Schneider *et al*, 2001). In patients with recurrent CNS GCTs, chemotherapy alone or combined with radiotherapy has to be considered, if feasible (Sawamura *et al*, 1999; Kellie *et al*, 2004).

In children with recurrent mediastinal or retroperitoneal primary GCT, the salvage strategy consists of chemotherapy followed by resection of residual masses, if necessary (Gobel *et al*, 2000). High-dose chemotherapy is currently investigated as a possible option for high-risk and recurrent CNS GCTs, and is used on an individual basis as salvage therapy for children with other extragonadal germ-cell malignancies (Calaminus and Patte, 2002; Cushing *et al*, 2004; Kellie *et al*, 2004; Modak *et al*, 2004).

Few series with salvage chemotherapy for relapse GCT patients with extragonadal primary are reported in literature. The largest experience with different salvage standard-dose chemotherapeutic approaches, more often including cisplatin-based chemotherapy, in relapse patients with sacrococcygeal GCT showed 10 of the 22 patients (45%) disease-free at a median followup of 56 months (Schneider *et al*, 2001). Other authors investigated the use of salvage treatments including standard-dose chemotherapy and/or HDC in patients with relapsed CNS GCT (Calaminus and Patte, 2002; Kellie *et al*, 2004). Overall, in these highly heterogeneous experiences, long-term remissions were obtained in nearly 25–50% of cases. Another report presented the largest series of patients with recurrent or progressive CNS GCTs treated with thiotepa-based HDC. No toxic deaths occurred. Out of the 21 patients, 11 (52%) achieved long-term disease-free survival. Results were clearly better for germinomas (seven of nine patients, 78%, disease-free continuously), than for nongerminomatous GCTs (four of 12 patients, 33%, long-term disease-free) (Modak *et al*, 2004). Recently, a regimen including high doses of cisplatin has been compared to standard-dose chemotherapy as primary treatment in a randomised study in 299 children with high-risk GCTs, including 165 extragonadal GCTs. Chemotherapy has consisted of bleomycin 15 U m^−2^, etoposide 500 mg m^−2^ and either cisplatin 200 mg m^−2^ (high-dose arm) or cisplatin 100 mg m^−2^ (standard-dose arm). The overall survival has been similar in both regimens; treatment-related deaths and grade 3–4 toxicities have been more common with HDC. As a consequence, the arm including high-dose cisplatin is not being recommend for paediatric patients as first-line treatment (Cushing *et al*, 2004).

In this report, we have presented the results of the EBMT experience with HDC as salvage treatment for children with extragonadal GCT. To the best of our knowledge, this is the largest reported experience with HDC in these patients as salvage setting. Only one patient with germinoma (pt. no. 4 in the tables) was included in this analysis. Nine patients received HDC in first- and 14 in second- or third-relapse situation. All extracranial GCT patients but one were chemosensitive before HDC, while two intracranial GCT patients were chemorefractory. With a median followup of 66 months (range, 31–173), of 23 extragonadal GCT patients who received salvage HDC, 10 (43%) are disease-free continuously. Since another patient with disease recurrence achieved a disease-free status after further chemotherapy, 11 extragonadal GCT patients (48%) are currently disease-free: eight of 14 patients (57%) with extracranial primary GCT, and three of nine patients (33%) with intracranial primary GCT. Therefore, HDC could be a possible option as salvage treatment for extracranial GCTs. Our results for intracranial GCTs, including only one germinoma, are similar to those of other major experiences for nongerminomatous CNS GCTs (Modak *et al*, 2004). The 2-year overall survival rates for first relapsing patients and second–third relapsing extragonadal GCT patients were clearly different: 78 and 43%, respectively. In our experience, HDC induces a high rate of long-term remissions even as third-line treatment. An induction regimen before HDC was given in nine patients, five (56%) are continuously disease-free. Multiple HDC courses were given in five cases as upfront salvage treatment, in four with PBPC support. Of these five children, three died of disease. Therefore, either an induction regimen before HDC or multiple upfront HDC courses were not associated with improved results (Table 3).

In the EBMT experience, no toxic deaths occurred, and the two cases of VOD were reported in the only two patients treated with the four-drug HDC regimen, including carboplatin, etoposide, thiotepa and melphalan. The tolerability of HDC could be related to the HDC regimens used. Most commonly, patients received carboplatin- or etoposide-based HDC regimens, and only in one case high-dose cisplatin was given (Table 2). Moreover, no patients developed myelodysplasia or secondary neoplasms after receiving HDC in our short experience, even though an increased risk of acute myelogenous leukaemia was reported in children with GCT after standard-dose chemotherapy (Schneider *et al*, 1999).

In conclusion, in the EBMT experience, HDC with HPCS induced impressive long-term remissions as salvage treatment in children with extragonadal extracranial GCTs. Salvage HDC should be investigated in prospective trials in these patients. New strategies should be considered for salvage treatment of patients with CNS GCT.

## Figures and Tables

**Table 1:** Patient characteristics at diagnosis and treatments before high-dose chemotherapy

**No.**	**Age**	**Sex**	**Primary location**	**Histology**	**Increased markers**	**Metastatic sites**	**Primary treatment**	**Response**
1	1	M	Sacr	EC, IT	AFP=794	Lung	RPTR; Cis, Vbl, Bl, VP16, Ifo (Adj)	CR
2	1	F	Sacr	NSGCT	AFP=22 250	Liver, B	RPTR; Cis, Vbl, Bl, VP16, Ifo, AcD, Cy; PRLM	PR+
3	13	M	CNS	Unknown	AFP=3710	None	Carbop, VP16, Ifo	PD
4	15	F	CNS	Germin	None	None	RT-CNS; Cis, Vbl, Bl, VP16, Ifo	CR
5	1	F	Sacr	NSGCT	AFP=54 290, LDH=931	None	PPTR; CT	PR+
6	1	F	Sacr	NSGCT	AFP=13 750	Bone	Cis, Vbl, Bl	CR
7	13	M	CNS	MT, YST	AFP=305	None	Carbop, VP16, Bl	PR+
8	14	M	CNS	Unknown	AFP/HCG exact values unknown	None	Cis, VP16, Bl; RT-CNS	CR
9	1	M	Sacr	YST	AFP=10 4000	Retr, Liver	Cis, Vcr, Ifo, Carbop, Vbl, Bl,	PR−
10	11	M	CNS	EC	AFP=43; HCG=142	None	RT-CNS; Cis, Vcr, VP16, Cy	PR−
11	13	M	Med	NSGCT	AFP=6100; HCG=920	None	Carbop, Vbl, Bl, AcD	PR+
12	6	F	Sacr	EC, YST	HCG=18 042; LDH=999	Med, CNS, Lung, B	Cis, Vcr, Mtx, Bl, AcD, Cy, VP16	PD
13	18	M	CNS	EC, MT	AFP=416; HCG=30; LDH=1149	None	RT-CNS	PR−
14	7	M	CNS	Unknown	AFP=145, HCG=53	None	RT-CNS	CR
15	1	F	Sacr	EC	AFP=46 100	None	PPTR; Carbop, Cy, AcD, Vbl, Bl	CR
16	9	M	Retr	NSGCT	Unknown	None	RPTR; Vcr, AcD (Adj)	CR
17	12	M	CNS	Unknown	AFP=339	None	CT; RT-CNS	PR+
18	1	F	Sacr	YST	AFP=30 947, LDH=828	Retr, Liver	Cis, Vbl, Bl, VP16, Ifo, AcD	CR
19	19	M	CNS	CC	HCG=40 400	Lung	CT; RT-CNS	PD
20	11	M	Med	EC, YST	None	None	RPTR; Cis, VP16, Bl (Adj)	CR
21	7	M	Retr	NSGCT	Unknown	None	PPTR; Cis, VP16, Bl	PR+
22	4	F	Retr	YST	AFP=27 8000, LDH=1786	Liver	Cis, Vbl, Bl, VP16, Ifo	CR
23	1	F	Retr	EC	AFP=14 8600	None	PPTR; Cis, VP16, Bl	PR+

M, male; F, female; Sacr, sacrococcix; CNS, central nervous system; Med, mediastinum; Retr, retroperitoneum; B, bone; IT, immature teratoma; MT, mature teratoma; YST, yolk sac tumour; EC, embryonal carcinoma; CC, choriocarcinoma; Germin, germinoma; NSGCT, nonseminomatous germ-cell tumour (not furthermore specified); AFP, alpha-fetoprotein (ng ml^−1^); HCG, human choriogonadotropin (IU l^−1^); RPTR, radical primary tumour resection; PPTR, partial primary tumour resection; PRLM, partial resection liver metastases; Cis, cisplatin; Vbl, vinblastin; Bl, bleomycin; VP16, etoposide; Ifo, ifosfamide; AcD, actinomycin D; Cy, cyclophosphamide; Carbop, carboplatin; Vcr, vincristine; Mtx, methotrexate; CT, chemotherapy (schedule/drugs not available); RT, radiotherapy; Adj, adjuvant; CR, complete remission; PR−, marker-negative partial remission; PR+, marker-positive partial remission; SD, stable disease; PD, progressive disease.

**Table 2:** High-dose chemotherapy regimens

**Abbreviation**	**Chemotherapeutic drugs**	**No. HDC courses**
CarboPEC	Carboplatin 250–350 mg m^−2^ × 4 days or dosed with Calvert formula with AUC=7 (lower dosed used)	
	Etoposide 250–400 mg m^−2^ × 4 days	7
	Cyclophosphamide 1.6 g m^−2^ × 4 days	
		
CE	Carboplatin 250–500 mg m^−2^ × 3–4 days or dosed with Calvert formula with AUC=7 (lower dosed used)	5
	Etoposide 250–400 mg m^−2^ × 3–4 days	
		
TE	Thiotepa 300 mg m^−2^ × 3 days	5
	Etoposide 250–300 mg m^−2^ × 3 days	
		
CarboPETM	Carboplatin 250–350 mg m^−2^ × 3–4 days or dosed with Calvert formula with AUC=7 (lower dosed used)	
	Etoposide 250–400 mg m^−2^ × 3–4 days	2
	Thiotepa 200–250 mg m^−2^ × 2–3 days	
	Melphalan 80–100 mg m^−2^ × 1 day	
		
Other regimens	HD-CyC=high-dose cyclophosphamide 7 g m^−2^ (*n*=2); CM=cyclophosphamide/melphalan (*n*=2); TM=thiotepa/melphalan (*n*=1); TC=thiotepa/cyclophasphamide (*n*=1); ICE=ifosfamide/carboplatin/etoposide (*n*=1); HD-PEC=cisplatin/etoposide/cyclophosphamide (*n*=1); CarboPTC=carboplatin/thiotepa/cyclophosphamide (*n*=1); not available (*n*=1)	

**Table 3:** Salvage high-dose chemotherapy for children with extragonadal germ-cell tumour

**No.**	**T. to first Rel/PD (mo)**	**Second-line standard-dose chemotherapy before HDC (response)**	**T. to second Rel/PD (mo)**	**HDC setting (no. Rel)**	**Induction regimen (response)**	**Status before HDC**	**Sites of disease before HDC**	**HDC regimen (no. cycles)**	**Response**	**Further therapies (response)**	**DFS (mo)**	**OS (mo)**
1	4	None	NA	1st Rel	Carbop, Vbl, Bl, Vcr, AcD, Adr, Cy(CR)	CR	NED	CarboPEC (1)	CR	Surg, CT, RT (CR)	21	173+
2	10	None	NA	1st Rel	None	SR	Liver, B	PEC (1), CE (1)	CR	None	77+	77+
3	NA	None	NA	1st Rel	None	PD	CNS	TE (1)	PT	RT-CNS (Unk)	0	12
4	11	Cis, VP16, Ifo, RT-CNS (CR)	19	2^nd^ Rel	None	SR	CNS	CE (1)	CR	RT-CNS (Adj)	73+	73+
5	9	None	NA	1st Rel	CT (PR–), STRR (CR)	CR	NED	CarboPEC (1)	CR	None	73+	73+
6	7	Cis, VP16, Ifo (PR–)	14	2nd Rel	Ifo, Adm (PR–)	PR–	Unk	TE (1)	PR−	RRRD (CR)	68+	68+
7	4	Carbopl, VP16, RT-CNS (SD)	2	2nd Rel	None	SD	CNS	HD-Cyc (2)	PD	CT (PD)	0	3
8	16	None	NA	1st Rel	Cis, Vcr, Ifo (CR)	CR	NED	CarboPETM (1)	CR	Unk (Unk)	Unk	51
9	7	Carbop, Vcr, AcD, Adr (PR+)	8	2nd Rel	None	PR+	Sacr, Retr, Liver	CarboPTC (1)	PD	None	0	5
10	5	None	NA	1st Rel	None	SR	CNS	CM (1)	CR	None	66+	66+
11	2	None	NA	1st Rel	VP16, Ifo (PR-), MTRR (CR)	CR	NED	TE (1)	CR	None	63+	63+
12	1	Cis, Vcr, Bl, VP16, Ifo (PD)	NA	2nd Rel	None	PD	Med, CNS, Lung, B	CarboPEC (1)	PD	None	0	3
13	2	Cis, VP16, Bl (PR−)	8	2nd Rel	RT-CNS, Cis, VP16, Ifo (PT)	PT	CNS	CarboPEC (1)	PT	Surg (CNS)	0	16
14	62	Cis, BCNU, VP16, Cy (PR−)	6	2nd Rel	None	SR	CNS	CM (1)	CR	RT-CNS, CT (PD)	5	5
15	9	Cis, VP16, Ifo (CR)	9	2nd Rel	None	SR	Unk	TE (1)	CR	None	63+	63+
16	4	Ifo, VP16, Vcr, AcD, Cy (CR)	41	2nd Rel	Carbopl, VP16, Ifo, Cy (CR)	CR	NED	CarboPETM (1)	CR	Unk (PD)	Unk	29
17	18	Carbop, VP16, Ifo, Cis, Vbl (PD)	NA	2nd Rel	None	PD	CNS	TE (1)	CR	None	40+	40+
18	8	Cis, VP16, Vcr (PR+)	6	2nd Rel	Carbop, Vbl, Bl (PT)	PT	Lung	CarboPEC (1)	CR	RT-Lung	9	11
19	1	Cis, VP16, Bl, Ifo (PR+)	3	2nd Rel	None	PR+	CNS	CarboPEC (2)	CR	RT-CNS, CT (PD)	6	8
20	9	None	NA	1st Rel	None	SR	Unk	ICE (1), TC (1)	PD	CT, Surg (PD)	0	15
21	3	Cis, Vcr, Mtx, Bl, AcD, Cy (PR+)	6^*^(5^**^)	3rd Rel	None	PR+	Retr	CE (3)	PR−	RRRD (CR)	34+	34+
22	26	None	NA	1st Rel	Carbop, VP16, Ifo (CR)	CR	NED	TM (1)	CR	None	31+	31+
23	10	Cis, VP16, Bl (PR+)	8	2nd Rel	None	PR+	Retr	Unk (1)	PR+	None	0	5

^*^After 6 months second Rel, treated with third-line standard-dose chemotherapy with Ifo,Vbl, VP16 (PR+); ^**^after 5 months 3rd Rel/PD.

Abbreviations: T.to first or second Rel/PD, Time to first or second relapse/progressive disease; HDC, high-dose chemotherapy; mo, months; NA, not applicable; Sacr, sacrococcix; CNS, central nervous system; Retr, retroperitoneum; Med, mediastinum; B, bone; NED, not evidence of disease; Unk, unknown; IT, immature teratoma; MT, mature teratoma; YST, yolk sac tumour; EC, embryonal carcinoma; Germ, germinoma; CC, choriocarcinoma; AFP, alpha-fetoprotein (ng ml^−1^); HCG, human choriogonadotropin (IU l^−1^); MTRR, mediastinal tumour radical resection; STRR, sacrococcigeal tumour radical resection; RRRD, radical resection of residual disease; Cis, cisplatin; Vbl, vinblastin; Bl, bleomycin; VP16, etoposide; Ifo, ifosfamide; ActD, actinomycin D; Cy, cyclophosphamide; Carbop, carboplatin; Vcr, vincristine; BCNU, bendamustine; Mtx, methotrexate; Adm, adriamycin; CT, chemotherapy (schedule/drugs not available); RT, radiotherapy; Adj, adjuvant; CR, complete remission; PR−, marker-negative partial remission; PR+, marker-positive partial remission; SD, stable disease; PD, progressive disease; PT, persistent tumour with response not evaluable; SR, sensitive relapse (indicating patients relapsing after a previous CR or PR− and receiving frontline HDC without induction regimens). For abbreviations of HDC regimens, see Table 2.

## Appendix

**The following institutions contributed to the study** S Slavin, D Engelhard Department of Bone Marrow Transplantation and Cancer Immunotherapy, Hadassah University Hospital, Jerusalem, Israel; I Yaniv, S Stein, Department of Paediatric Haematology Oncology, Schneider Children's Medical Center of Israel, Petah Tikva, Israel; JL Harousseau, Department of Haematology, University Hospital, Nantes, France; A Tobler, K Leibundgut, M Fey, Division of Haematology/Oncology, University Hospital, Bern, Switzerland; D Bron, Experimental Haematology, Institut Jules Bordet, Brussels, Belgium; V Castel, Department of Paediatric Oncology, Hospital Infantil La Fe, Valencia, Spain; J Cornish, Department of Paediatric Oncology/BMT, Royal Hospital for Children, Bristol, UK; H Gadner, R Ladenstein, Department of Haematology and Oncology, St Anna Childrens Hospital, Vienna, Austria; S McCann Shaun McCann, Department of Haematology and Institute for Molecular Medicine St James's Hospital and University of Dublin, Trinity College, Dublin, Ireland; H Joensuu, Department of Oncology, University of Helsinki, Finland; C Mitchell, Paediatric Oncology, Oxford Radcliffe Hospital, Oxford, UK; E Plouvier, Unite d’haematologie infantile, Hopital Saint-Jacques, CHU, Besancon, France; R Schots, BMT Unit, Academisch Ziekenhuis-Vrije Universiteit Brussel, Brussels, Belgium; J-P Vannier, Service d’Immuno-Haemato-Oncologie Paediatrique, Centre Hospitalier Universitaire, Rouen, France; A Yalcin, Department of Haematology, Gulhane Military Medical Academy, Ankara, Turkey; MA Yesilipek, Department of Paediatric Haematology and Immunology, Akdeniz University School of Medicine, Antalya, Turkey.
